# Effects of Ketamine on Postoperative Pain After Remifentanil-Based Anesthesia for Major and Minor Surgery in Adults: A Systematic Review and Meta-Analysis

**DOI:** 10.3389/fphar.2018.00921

**Published:** 2018-08-17

**Authors:** Juan F. García-Henares, Jose A. Moral-Munoz, Alejandro Salazar, Esperanza Del Pozo

**Affiliations:** ^1^Hospital Marina Salud, Dénia, Alicante, Spain; ^2^Department of Nursing and Physiotherapy, University of Cádiz, Cádiz, Spain; ^3^Institute of Research and Innovation in Biomedical Sciences of the Province of Cadiz (INiBICA) University of Cádiz, Cádiz, Spain; ^4^Preventive Medicine and Public Health Area, University of Cádiz, Cádiz, Spain; ^5^The Observatory of Pain (External Chair of Pain), University of Cádiz, Cádiz, Spain; ^6^Department of Pharmacology, Faculty of Medicine, Institute of Neurosciences, Biomedical Research Institute Granada, University of Granada, Granada, Spain

**Keywords:** remifentanil, ketamine, minor surgery, mayor surgery, NMDA antagonist, meta-analysis

## Abstract

Ketamine, an N-methyl-D-aspartate (NMDA) receptor antagonist, has been postulated as an adjuvant analgesic for preventing remifentanil-induced hyperalgesia after surgery. This systematic review and meta-analysis aims to assess the effectiveness of ketamine [racemic mixture and *S*-(+)-ketamine] in reducing morphine consumption and pain intensity scores after remifentanil-based general anesthesia. We performed a literature search of the PubMed, Web of Science, Scopus, Cochrane, and EMBASE databases in June 2017 and selected randomized controlled trials using predefined inclusion and exclusion criteria. To minimize confounding and heterogeneity, studies of NMDA receptor antagonists other than ketamine were excluded and the selected studies were grouped into those assessing minor or major surgery. Methodological quality was evaluated with the PEDro and JADA scales. The data were extracted and meta-analyses were performed where possible. Twelve RCTs involving 156 adults who underwent minor surgery and 413 adults who underwent major surgery were included in the meta-analysis. When used as an adjuvant to morphine, ketamine reduced postoperative morphine consumption in the first 24 h and postoperative pain intensity in the first 2 h in the minor and major surgery groups. It was also associated with significantly reduced pain intensity in the first 24 h in the minor surgery group. Time to the first rescue analgesia was longer in patients who received ketamine and underwent major surgery. No significant differences in the incidence of ketamine-related adverse effects were observed among patients in the intervention group and controls. This systematic review and meta-analysis show that low-dose (≤0.5 mg/kg for iv bolus or ≤5 μg/kg/min for iv perfusion) of ketamine reduces postoperative morphine consumption and pain intensity without increasing the incidence of adverse effects.

## Introduction

Management of chronic postsurgical pain remains a challenge for anesthesiologists and surgeons. Approximately 240 million surgical procedures are performed worldwide each year, and an estimated 12% of patients still report moderate to intense pain 1 year after surgery (Fletcher et al., 2015). Inadequately treated pain could be considered an adverse postoperative effect, as it can lead to longer hospital stays, higher costs, and lower patient satisfaction (Shipton, 2014). Acute postoperative pain is also a risk factor for the development of chronic postsurgical pain (Perkins and Kehlet, 2000), and the relationship appears to be directly proportional, with more intense or longer-lasting pain linked to a higher incidence of chronic pain (Mion and Villevieille, 2013; Pozek et al., 2016; Reddi, 2016).

Remifentanil is a widely used general anesthesia thanks to its pharmacodynamic and pharmacokinetic properties (Kim et al., 2015). Its potency as an opioid agonist combined with a short elimination half-life without accumulating with prolonged infusion allows anesthesiologists to maintain hemodynamic stability during surgery without risk of delayed awakening. Intraoperative remifentanil infusion has, however, been associated with opioid-induced hyperalgesia (Joly et al., 2005; Fletcher and Martinez, 2014).

The N-methyl-D-aspartate (NMDA) receptor is believed to play an important role in the pathophysiology of opioid-induced hyperalgesia (Mao et al., 1995; Mayer et al., 1999; Williams et al., 2001; Ossipov et al., 2005; Angst and Clark, 2006; Mao, 2006). Ketamine is a non-competitive NMDA receptor antagonist (Mion and Villevieille, 2013) authorized by the U.S. Food and Drug Administration as an anesthetic drug and it is used in multimodal analgesia regimens to improve postoperative pain control (Chou et al., 2016). The classic anesthetic effect of ketamine is described as a dose-dependent central nervous system (CNS) depression that leads to a dissociative state characterized by profound analgesia and amnesia but not necessarily loss of consciousness (Kohrs and Durieux, 1998). The use of low subanesthetic doses of ketamine (no more than 1 mg/kg iv bolus or 20 μg/kg/min continuous infusion) as an adjuvant to morphine in postoperative multimodal analgesia regimens (Schmid et al., 1999) is supported by several lines of evidence: (i) the mechanism of action of ketamine and the importance of the NMDA neurotransmission system in nociceptive processing (Bell et al., 2015); (ii) evidence that ketamine potentiates the analgesic effects of opioids, suggesting that it could reduce acute postoperative pain and minimize opioid consumption (Carstensen and Möller, 2010; Song et al., 2013); and (iii) the low toxicity of subanesthetic doses of ketamine (Michelet et al., 2007). In addition, recent studies have attributed additional antihyperalgesic, neuroprotective, antidepressant, and anti-inflammatory effects to ketamine based on its interaction with multiple other receptors, such as AMPA, kainate, gamma-aminobutyric acid (GABA), opiate, muscarinic, as well as voltage-gated sodium and hyperpolarization-activated cyclic nucleotide-gated channels (Zanos et al., 2018). The isomer *S*(+)-ketamine (also named Esketamine) is reported to be twice as potent as the racemic mixture as an anesthetic (Zanos et al., 2018). Despite these seemingly “ideal” qualities of ketamine, however, contradictory results have been reported for the efficacy of ketamine in multimodal perioperative analgesia regimens.

We wondered if there was clinical evidence supporting the use of perioperative ketamine to improve postoperative pain after remifentanil-based anesthesia in adults. To our knowledge, only two meta-analyses, each analyzing 14 randomized controls trials (RCTs), have been conducted (Liu et al., 2012; Wu et al., 2015) and they reported conflicting findings One of the analyses found no significant evidence that NMDA antagonists prevented remifentanil-associated hyperalgesia (Wu et al., 2015), while the other one showed that ketamine significantly reduced postoperative pain and cumulative morphine consumption (Wu et al., 2015). This benefit was also found in a meta-analysis of the addition of ketamine to morphine in patient-controlled analgesia devices (Assouline et al., 2016). The contradictory results could be partly due to the fact that the meta-analyses did not distinguish between different types of surgery.

The aim of this systematic review and meta-analysis was to evaluate the influence of perioperative ketamine within a multimodal analgesia regimen in adults undergoing surgery, distinguishing between minor and major procedures and excluding studies of NMDA antagonists other than ketamine to minimize confounding and heterogeneity.

## Materials and methods

We performed a systematic review of the literature in accordance with the PRISMA (Preferred Reporting Items for Systematic Reviews and Meta-Analyses) statement.

### Eligibility criteria

An initial database search was undertaken to identify RCTs examining the use of perioperative low-dose ketamine in remifentanil-based general anesthesia for major or minor surgery. Only studies that were performed in adults and used morphine as a postoperative analgesic were eligible for inclusion. RCTs reporting on postoperative cumulative morphine consumption, pain intensity, pain outcomes, and adverse opioid or ketamine effects were considered.

### Information sources and search

A search of the PubMed, Web of Science, Scopus, and Cochrane databases was performed in June 2017. Only articles written in English or Spanish were included. The search queries used for each database are shown in Table 1.

**Table 1:** Search strategies and results in each bibliographic database.

**Database**	**Search Query**	**Results**
PubMed	((“Remifentanil” [Supplementary Concept]) AND “Ketamine”[Mesh]) AND “Hyperalgesia”[Mesh]	19
Cochrane	Remifentanil AND Ketamine AND Hyperalgesia Refined by: Document Types: Trials	36
Web of science	TS = (Remifentanil AND Ketamine AND Hyperalgesia) Refined by: Databases: Core Collection AND Document Types: Clinical Trials	57
Scopus	TITLE-ABS-KEY (Remifentanil AND Ketamine AND Hyperalgesia) Refined by: Document Types: Articles	58

### Study selection

Two reviewers (JFGH and JAMM) independently performed the search and assessed the suitability of the articles for inclusion. In the event of disagreement, the reviewers discussed the discrepancies and decided whether or not to include the article. If they were unable to reach an agreement, a third reviewer (AS) was involved. In a pre-selection phase, the abstracts of all the articles retrieved by the literature search were screened for eligibility. Potentially eligible studies were then assessed in depth by examining the full text prior to inclusion.

### Data collection process

Relevant data from selected articles were extracted and recorded in a purpose-designed spreadsheet by a single author (JFGH) using a standardized procedure. The authors of one original article that met the inclusion criteria but had some missing information on means and standard deviation were contacted twice by e-mail, but they did not reply (Hong et al., 2011). The information extracted by JFGH was independently reviewed by two authors (JAMM and AS).

### Data items

The following data were extracted from each study: study design, study population, ketamine and remifentanil regimens, description of the intervention or control and experimental groups, type of surgical procedure, postoperative analgesia strategies, follow-up period, and outcome measures (Table 2).

**Table 2:** Details of the selected studies.

**Author**	**Year**	**Sample size (K/control)**	**Ketamine protocol**	**Remifentanil infusion rate**	**Procedure**	**N20**	**Anesthesia maintenance**	**Postoperative analgesia**
Aubrun et al.	2008	45/45	0.5 mg/kg iv before surgical incisión + 5 mg/ml postoperative PCA	0.5 μg/kg/min	Gynecological surgery	NO	Propofol	Morphine PCA
Ganne et al.	2005	30/31	0.15 mg/kg iv + 2 μg/kg/min	0.25 μg/kg/min	ENT surgery	NO	Propofol	Morphine PCA+ Paracetamol 1 g/6 h+ methylprednisolone 2 mg/kg/dia
Guignard et al.	2002	25/25	0.15 mg/kg iv + 2 μg/kg/min	0.25 μg/kg/min	Open colorrectal surgery	NO	Desfluorane	Morphine PCA
Hadi et al.	2013	30/15	1 μg/kg/min (±1 μg/kg/min postoperative)	0.2 μg/kg/min	Lumbar microdiscectomy	Yes	Sevofluorane	Morphine PCA
Hadi et al.	2010	15/15	1 μg/kg/min	0.2 μg/kg/min	Spinal fusion	YES	Sevofluorane	Morphine Pump
Jaksch et al.	2002	15/15	0.5 mg/kg iv + 2 μg/kg/min	0.5 μg/kg/min	Arthroscopic ACL repair	NO	Propofol	Morphine PCA
Joly et al.	2005	24/25	0.5 mg/kg iv +5 μg/kg/min+ 2 μg/kg/min postoperative infusion	0.4 μg/kg/min	Abdominal surgery	NO	Desfluorane	Morphine PCA
Leal et al.	2015	28/28	5 μg/kg/min	0.4 μg/kg/min	Laparoscopic cholecystectomy	NO	Isoflourane	Morphine PCA
Lee et al.	2014	20/20	0.3 mg/kg+ 3 μg/kg/min	4 ng/ml	Laparoscopic cholecystectomy	NO	Sevofluorane	Morphine PCA
Sahin et al.	2004	17/16	0.5 mg/kg iv	0.1 μg/kg/min	Lumbar disk operation	YES	Desfluorane	Morphine PCA
Van Elstraete	2004	20/20	0.5 mg/kg iv + 2 μg/kg/min	0.25 μg/kg/min	Tonsillectomy	NO	Propofol	Morphine i.v.
Yalcin et al.	2012	30/30	0.5 mg/kg	0.4 μg/kg/min	Total abdominal hysterectomy	NO	Desfluorane	Morphine PCA

Primary endpoints were cumulative morphine consumption (mg) in the first 24 h and pain intensity at 0, 2, 4, 12, and 24 h. Pain intensity was statistically standardized on a 0–10 cm visual analog scale (VAS). Due to the small number and heterogeneous nature of the articles selected, there were only two secondary endpoints: time to the first rescue analgesia and presence of ketamine or opioid adverse effects. Patient satisfaction and psychotic adverse effects in minor surgery were excluded from the meta-analysis as we were unable to obtain the missing data from the authors.

### Risk of bias

The methodological quality of the RCTs was analyzed using the PEDro (Maher et al., 2003) and Jadad (Clark et al., 1999) scales. The PEDro scale is an 11-item scale that assesses (i) notification of selection criteria, (ii) allocation of subjects to groups at random, (iii) concealment of allocation, (iv) similarity among groups at baseline in relation to the most important prognostic indicators, (v) blinding of participants, (vi) blinding of researchers/therapists, (vii) blinding of researchers measuring at least one key outcome, (viii) proportion of initial participants that contribute measures to the key results, (ix) compliance of the intervention assigned by the participants, (x) presentation of statistical comparisons between the groups, and (xi) presentation of specific measures and variability of the key results. One point is assigned to each criterion, except for (i), which is not included in the final score. The total possible score thus ranges from 0 to 10. Scores of 9 to 10 indicate excellent quality, 6to 8 good quality, 4 to 5 fair quality, and <4 poor quality (Gordt et al., 2018). With the Jadad scale, studies receive a score of 0–5 points (with higher scores representing higher methodological quality) depending on whether they (i) are described as randomized or doubled blind, (ii) use an appropriate randomization sequence or blinding procedure, and (iii) provide detailed information on withdrawals and dropouts. As studies of low methodological quality may overestimate treatment benefits (Moher et al., 1998), we only included studies with a PEDro score of 6 or higher (Clark et al., 1999) and a Jadad score of 3 or higher (Kang et al., 2018; Annex 1 in Supplementary Material).

### Statistical analysis

The 12 RCTs were grouped into 16 subgroups according to effect size and type of surgery (major or minor) (Table 3). A study could belong to more than one group if it reported more than one effect size or it analyzed both major and minor surgery. Studies could also appear more than once in the same subgroup if they performed comparisons with different groups under the same conditions.

**Table 3:** Characteristics of subgroups included in the meta-analysis.

**Subgroup**	**Trials included**	**Effect size**	**Heterogeneity test**	**Model**	Publication bias^*^
1	Van Elstraete et al., −30 min 2004 Leal et al., −30 min 2015 Van Elstraete et al., −1 h 2004 Leal et al., −1 h 2015 Van Elstraete et al., −2 h 2004 Leal et al., −2 h 2015	VAS 0–2 h Minor surgery	Heterogeneity *Q* = 225.2737; *df* = 5; *p* < 0.001	Random effects	No bias *Z* = 1.5029; *p* = 0.1329 *T* = −1.3473; *p* = 0.2492
2	Assouline et al., −1 h (2002) Jaksch et al., −2 h (2002) Aubrun et al., 0–30 min (2008)	VAS 0–2 h Major surgery	Homgeneity *Q* = 1.1184; *df* = 2; *p* = 0.5717	Fixed effects	No bias *Z* < 0.001; *p* > 0.999 *T* = 1.2596; *p* = 0.4272
3	Van Elstraete et al., 2004 Leal et al., 2015	VAS4 h Minor surgery	Heterogeneity *Q* = 14.7884; *df* = 1; *p* = 0.001	Random effects	–
4	Joly et al., 2005 Ganne et al., 2005 Aubrun et al., 2008	VAS 4 h Major surgery	Heterogeneity *Q* = 21.5624; *df* = 2; *p* < 0.001	Random effects	No bias *Z* < 0.001; *p* > 0.999 *T* = −0.6603; *p* = 0.6285
5	Van Elstraete, 2004 Hadi et al., 2013 Hadi et al., 2013 Leal et al., 2015	VAS 12 h Minor surgery	Heterogeneity *Q* = 104.3763; *df* = 3; *p* < 0.001	Random effects	Bias *Z* = 1.6984; *p* = 0.0894 *T* = −7.5979; *p* = 0.0169
6	Joly et al., 2005 Ganne et al., 2005 Aubrun et al., 2008	VAS 12 h Major surgery	Heterogeneity *Q* = 81.4968; *df* = 2; *p* < 0.001	Random effects	No bias *Z* = 1.0445; *p* = 0.2963 *T* = 2.0911; *p* = 0.2840
7	Van Elstraete, 2004 Hadi et al., 2013 Hadi et al., 2013 Leal et al., 2015	VAS 24 h Minor surgery	Heterogeneity *Q* = 197.5201; *df* = 3; *p* < 0.001	Random effects	No bias *Z* = 1.6984; *p* = 0.0894 *T* = −2.1538; *p* = 0.1641
8	Joly et al., 2005 Ganne et al., 2005 Aubrun et al., 2008	VAS 24 h Major surgery	Heterogeneity *Q* = 71.2937; *df* = 2; *p* < 0.001	Random effects	No bias *Z* = 1.0445; *p* = 0.2963 *T* = 3.5866; *p* = 0.1731
9	Van Elstraete, 2004 Hadi et al., 2013 Hadi et al., 2013 Leal et al., 2015	Morphine consumption minor surgery	Heterogeneity *Q* = 118.7848; *df* = 3; *p* < 0.001	Random effects	Bias *Z* = 1.6984; *p* = 0.0894 *T* = −16.5343; *p* = 0.0036
10	Sahin et al., 2004 Ganne et al., 2005 Aubrun et al., 2008 Hadi et al., 2010 Guignard et al., 2002 Yalcin et al., 2012	Morphine consumption major surgery	Heterogeneity *Q* = 326.9692; *df* = 5; *p* < 0.001	Random effects	Bias *Z* = 1.5029; *p* = 0.1329 *T* = −5.3088; *p* = 0.0061
11	Van Elstraete, 2004 Hadi et al., 2013 Hadi et al., 2013	Time to first rescue analgesia Minor surgery	Heterogeneity *Q* = 105.1229; *df* = 2; *p* < 0.001	Random effects	Bias *Z* = 1.0445; *p* = 0.2963 *T* = 419.7603; *p* = 0.0015
12	Jaksch et al., 2002 Guignard et al., 2002 Sahin et al., 2004 Joly et al., 2005	Time to first rescue analgesia Major surgery	Heterogeneity *Q* = 225.8723; *df* = 3; *p* < 0.001	Random effects	No bias *Z* = 0.3397; *p* = 0.7341 *T* = 3.6662; *p* = 0.0670
13	Van Elstraete, 2004 Hadi et al., 2013 Hadi et al., 2013 Leal et al., 2015	PONV Minor surgery	Heterogeneity *Q* = 3.9975; *df* = 3; *p* = 0.2617	Random effects	No bias *Z* = −0.3397; *p* = 0.7341 *T* = −0.2830; *p* = 0.8038
14	Guignard et al., 2002 Jaksch et al., 2002 Joly et al., 2005 Ganne et al., 2005 Aubrun et al., 2008	PONV Major surgery	Homogeneity *Q* = 1.7537; *df* = 4; *p* = 0.7809	Fixed effects	No bias *Z* = 0.7348; *p* = 0.4624 *T* = 1.0131; *p* = 0.3856
15^**^	Van Elstraete, 2004 Hadi et al., 2013 Hadi et al., 2013 Leal et al., 2015	Psychotic events minor surgery	–	–	–
16	Jaksch et al., 2002 Aubrun et al., 2008	Psychotic events major surgery	Homogeneity *Q* = 0.1108; *df* = 1; *p* = 0.7393	Fixed effects	–

* *Results not shown for subgroups with only two studies. PONV, postoperative nausea and vomiting; VAS, visual analog scale*.

** *Results not shown for subgroup 15, which was not included in the meta-analysis*.

The effect sizes considered were pain intensity measured on a 10-point VAS, where 0 indicated no pain and 10 indicated the worst possible pain; morphine consumption (mg); time to first rescue with analgesia (minutes); incidence of postoperative nausea and vomiting (PONV); and incidence of ketamine-related adverse events. All the RCTs compared the administration of ketamine and morphine (intervention group) vs. morphine only (control group).

A meta-analysis was carried out in each subgroup to compare the effect size between the intervention and control group. Standardized mean differences (SMDs) and 95% confidence intervals (CIs) were used for continuous variables (VAS, morphine consumption, and time to first rescue analgesia), while relative risk (RR) and 95% CIs were used for the incidence of PONV and psychotic events (anxiety, visual disturbances, impairment of cognitive functioning or florid psychotics symptoms like delirium or hallucinations). The significance level was set at *p* < 0.05.

Heterogeneity was determined using the Dersimonian and Laird test and the Cochran Q statistic. A fixed-effects model was used for studies with homogeneity and a random-effects model was used when there was significant heterogeneity. The latter accounts for variability due to differences between studies. The results of the meta-analyses are shown in forest plots. The plots show the differences between the intervention and control groups for mean values, and RRs, showing overall measures, together with the corresponding confidence intervals. Publication bias was analyzed (only in subgroups with three or more studies) using the Begg test (Z statistic) and the Egger test (t statistic). Finally, a sensitivity analysis was carried out to study the contribution of each study to the overall effect estimate. The analyses were carried out using the statistical software program EPIDAT 3.1. (Table 3).

## Results

### Study selection

**Figure 1:**
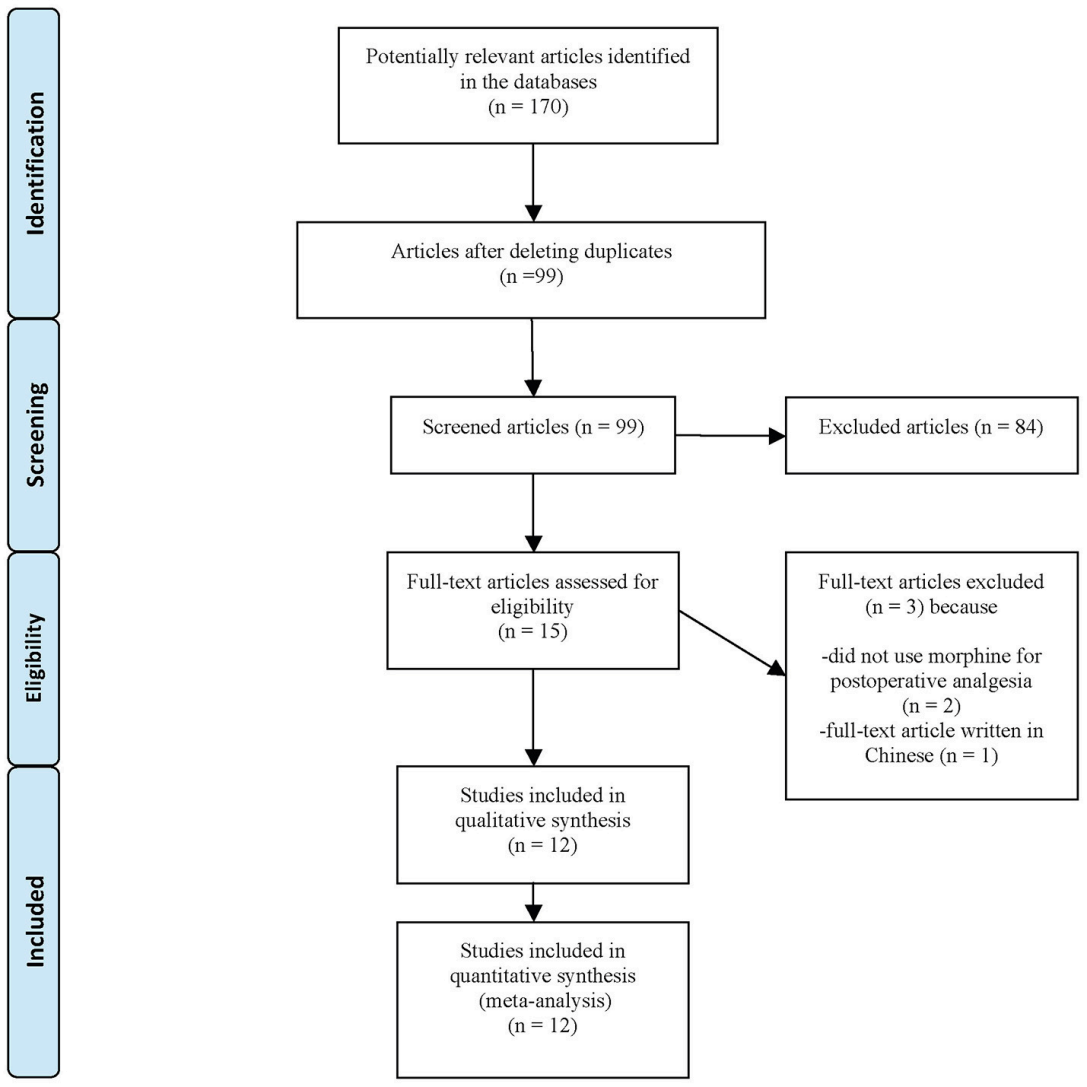
Flow diagram of the different phases of the systematic review.

The preliminary search identified 170 articles including 15 RCTs potentially responding to the inclusion criteria (Figure 1). Two of the RCTs were excluded because morphine was not used for postoperative analgesia (Launo et al., 2004; Choi et al., 2015) and one was excluded because the manuscript was written in Chinese (Launo et al., 2004). Twelve RCTs involving 569 adults were therefore included in the systematic review.

The effects of ketamine and remifentanil-induced hyperalgesia have been analyzed in two relatively recent meta-analyses: one by Liu et al. (2012) in 2012 and another by Wu et al. (2015) in 2015. Compared with Liu et al. (2012), we studied five additional RCTs and excluded seven (Liu et al., 2012), while compared with Wu et al. (2015), we studied four additional RCTs and excluded six (Wu et al., 2015). The studies were excluded because they included children, NMDA receptor antagonists other than ketamine (magnesium sulfate and amantadine), or postoperative opioids other than morphine.

### Study characteristics

The 12 RCTs were published between 2002 and 2015 (Table 2) and had been conducted in six countries: France (Moher et al., 1998; Clark et al., 1999; Maher et al., 2003; Launo et al., 2004; Ganne et al., 2005; Joly et al., 2005; Yu et al., 2005; Choi et al., 2015; Gordt et al., 2018; Kang et al., 2018), Austria (Jaksch et al., 2002), Jordan (Hadi et al., 2010, 2013), Brazil (Leal et al., 2015), Korea (Lee et al., 2014), and Turkey (Sahin et al., 2004; Yalcin et al., 2012). Nine RCTs involving 413 patients were assigned to the major surgery group while three involving 156 patients were assigned to the minor surgery group. Although all 12 RCTs used morphine to control postoperative pain, the administration regimens varied. Eight studies administered morphine via a patient-controlled analgesia system (Schmid et al., 1999; Jaksch et al., 2002; Sahin et al., 2004; Joly et al., 2005; Hadi et al., 2010, 2013; Kim et al., 2014; Lee et al., 2014; Roeckel et al., 2016; one of these also used paracetamol, Ganne et al., 2005) and two did not specify the type of pump (Reddi, 2016) or infusion system used (Van Elstraete et al., 2004).

### Synthesis of results

We were unable to assess the incidence of psychotic events in minor surgery (subgroup 15) by meta-analysis because only one of the studies yielded a result other than 0. The characteristics of the 16 subgroups are shown in Table 3, together with the results of the heterogeneity and publication bias tests. Subgroups 2, 14, and 16 were homogeneous and the rest were heterogeneous. Risk of publication bias was detected in subgroups 5, 9, 10, and 11, and their results should, therefore, be interpreted with caution. The results of the meta-analysis are summarized in Table 4.

**Table 4:** Meta-analysis results.

**Subgroup**	**Results**				**Forest plot**
	**Study**	***n***	**Mean difference (95% CI)**	**Weight (%)**	
VAS 0–2 h Minor surgery	Van Elstraete, −30 min (2004)	40	−7.83 (−9.65; −6.0027)	16.7362	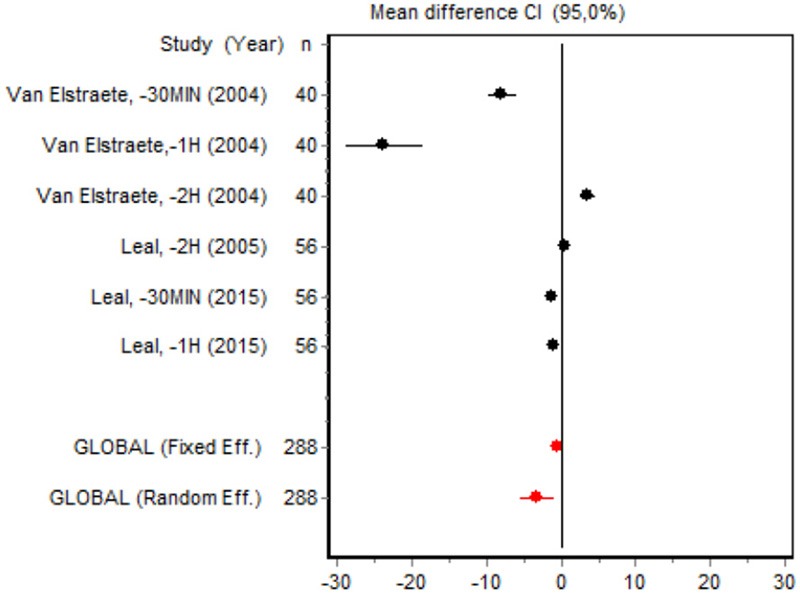
	Van Elstraete, −1 h (2004)	40	−23.5538 (−28.752218.3553)	9.3902	
	Van Elstraete, −2 h (2004)	40	3.6858 (2.667; 4.7038)	18.1041	
	Leal, −2 h (2015)	56	0.5411 (0.0078; 1.0744)	18.6037	
	Leal, −30 min (2015)	56	−1.2014 (−1.7706; 0.6323)	18.5768	
	Leal, −1 h (2015)	56	−0.9581 (−1.5112; −0.4051)	18.5890	
	Random effects	288	−3.1549 (−5.4066; −0.9033)		
VAS 0–2 h Major surgery	Jaksch, −1 h (2002)	30	−0.4071 (−1.1302; 0.3159)	21.0181	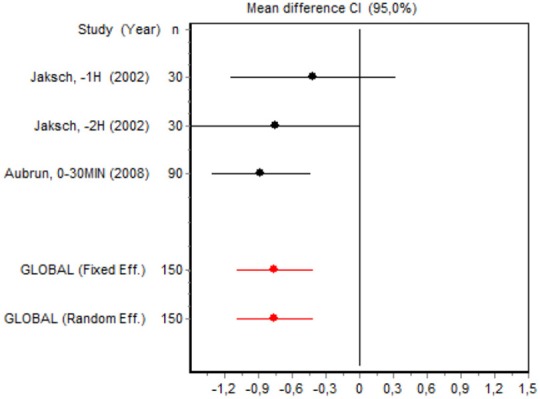
	Jaksch, −2 h (2002)	30	−0.7381 (−1.4778; 0.0015)	20.0857	
	Aubrun, 0–30 min (2008)	90	−0.8615 (−1.2935; 0.4296)	58.8962	
	Fixed effects	150	−0.7412(−1.0727; −0.4098)		
VAS 4 h Minor surgery	Van Elstraete, 2004	40	−2.1788 (−2.9612; 1.3964)	31.8379	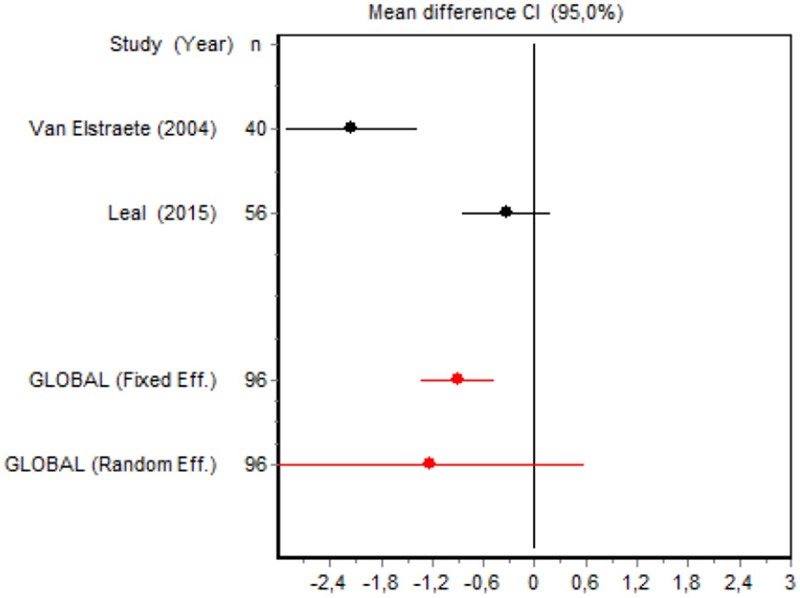
	Leal et al., 2015	56	−0.3301 (−0.8575; 0.1973)	34.5329	
	Random effects	96	−1.2309 (−3.0421; 0.5802)		
VAS 4 h Mayor surgery	Joly et al., 2005	49	−1.7351 (−2.3922; 1.0781)	31.7254	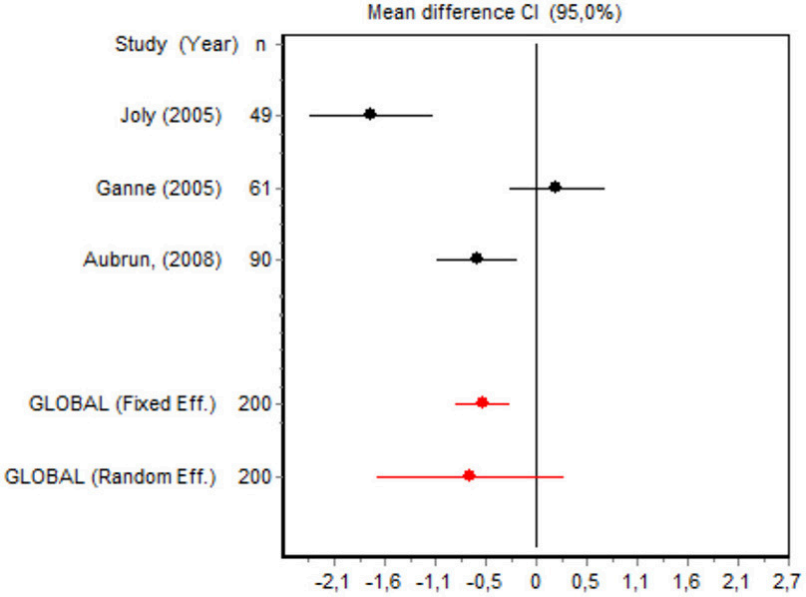
	Ganne et al., 2005	61	0.2193 (−0.2841; 0.7228)	33.6892	
	Aubrun et al., 2008	90	−0.6174 (−1.0403; 0.1945)	34.5854	
	Random effects	200	−0.6901 (−1.6751; 0.2948)		
VAS 12 h Minor surgery	Van Elstraete, 2004	40	−0.6143 (−1.2485; 0.0199)	27.2404	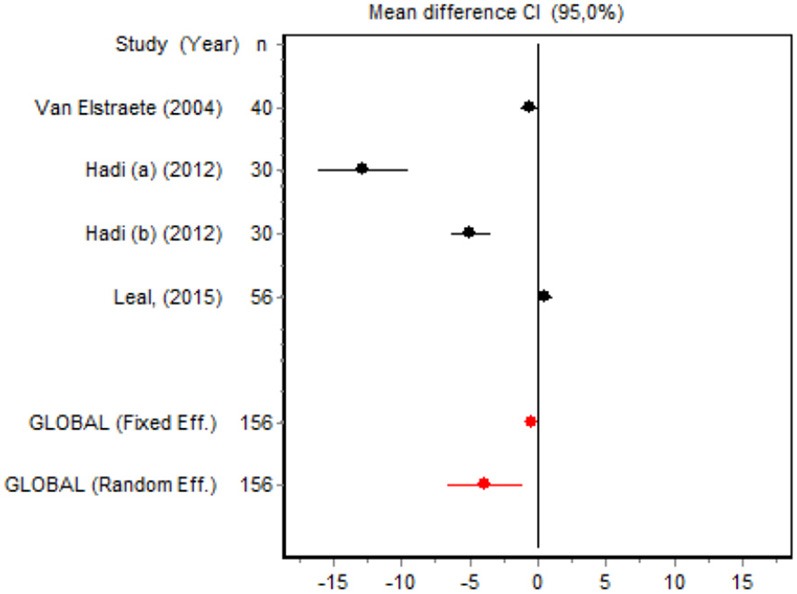
	Hadi et al., 2013	30	−12.7851 (−16.0984; −9.4719)	19.7066	
	Hadi et al., 2013	30	−4.9236 (−6.3604; −3.4869)	25.6965	
	Leal et al., 2015	56	0.5562 (0.0224; 1.0901)	27.3564	
	Random effects	156	−3.7999 (−6.5450; −1.0548)		
VAS 12 h Major surgery	Joly et al., 2005	49	2.3720 (1.6410; 3.1029)	32.8124	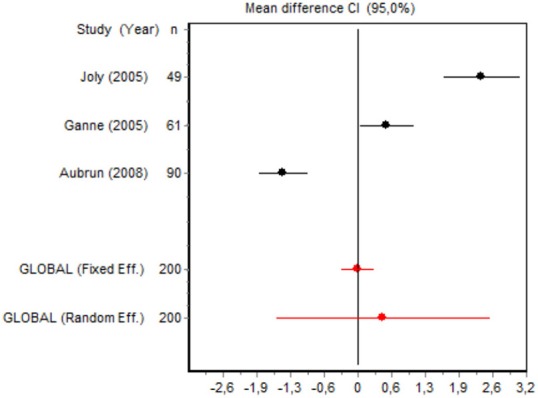
	Ganne et al., 2005	61	0.5523 (0.0408; 1.0637)	33.5299	
	Aubrun et al., 2008	90	−1.4282 (−1.8910; −0.9653)	33.6576	
	Random effects	200	0.4828 (−1.5621; 2.5276)		
VAS 24 h Minor surgery	Van Elstraete, 2004	40	−2.6816 (−3.5357; −1.8275)	26.1858	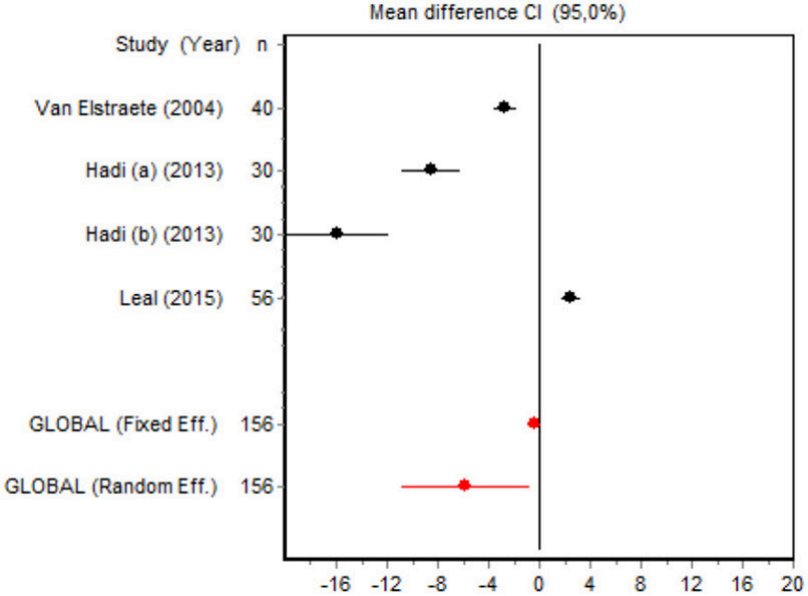
	Hadi et al., 2013	30	−15.8746 (−19.9546; −11.7946)	22.5099	
	Hadi et al., 2013	30	−8.4976 (−10.7637–6.2315)	25.9533	
	Leal et al., 2015	56	2.4906 (1.7926; 3.1886)	26.2510	
	Random effects	156	−5.7507 (−10.8028; −0.6986)		
VAS 24 h Major surgery	Joly et al., 2005	49	2.5823 (1.8239; 3.3407)	32.6371	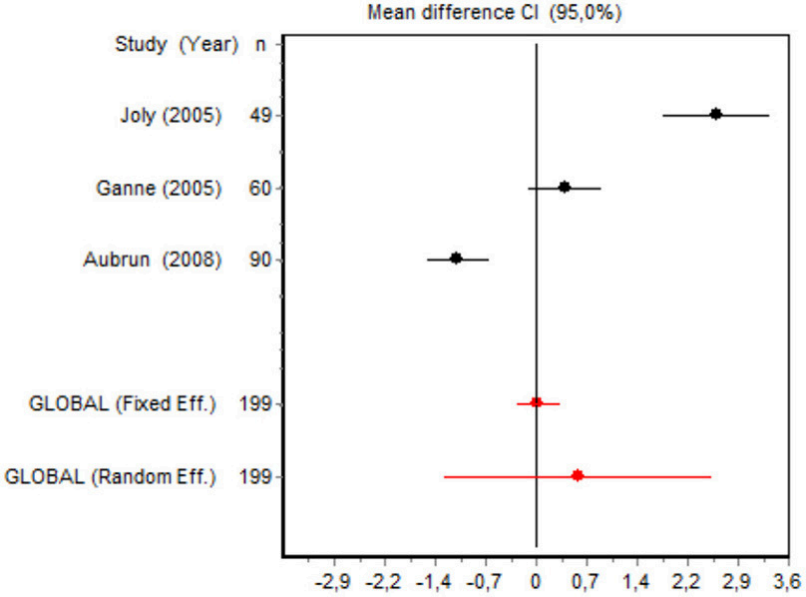
	Ganne et al., 2005	60	0.4164 (−0.0951; 0.9279)	33.5812	
	Aubrun et al., 2008	90	−1.1166 (−1.5609; −0.6724)	33.7817	
	Random effects	199	0.6054 (−1.3021; 2.5130)		
Morphine consumption minor surgery	Van Elstraete, 2004	40	−1.0657 (−1.7280; −0.4033)	35.1136	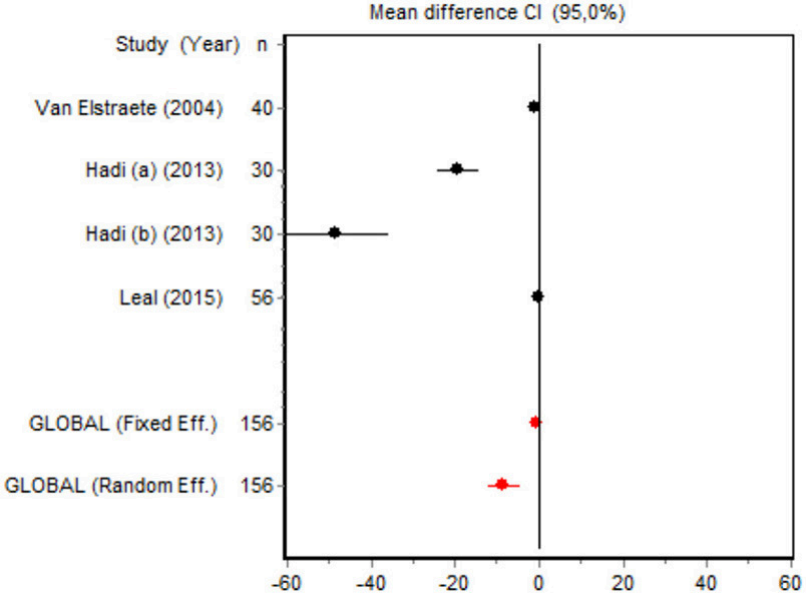
	Hadi et al., 2013	30	−19.3089 (−24.2468; −14.3710)	22.1079	
	Hadi et al., 2013	30	−48.2743 (−60.5101; −36.0385)	7.5228	
	Leal et al., 2015	56	−0.1003 (−0.6244; 0.4239)	35.2556	
	Random effects	156	−8.3099 (−12.0904; −4.5295)		
Morphine consumption major surgery	Sahin et al., 2004	33	1.0361 (0.3091; 1.7631)	17.3244	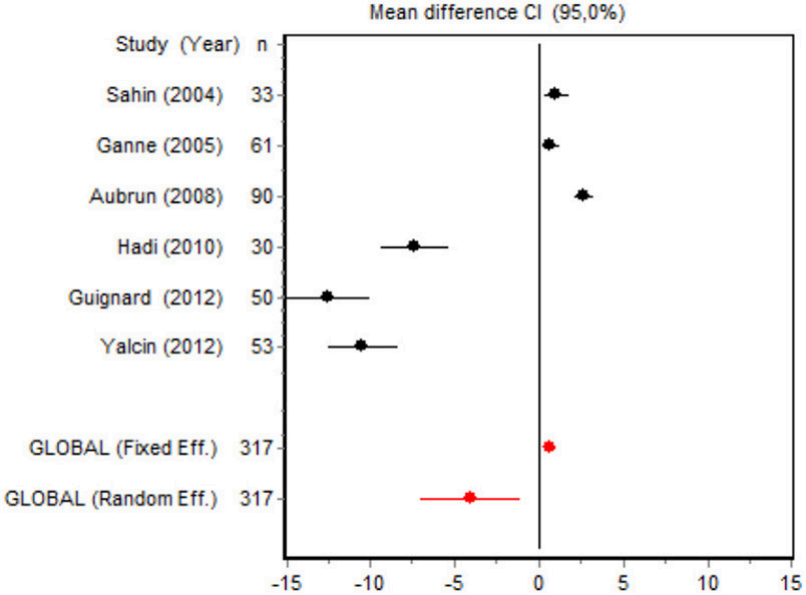
	Ganne et al., 2005	61	0.6916 (0.1748; 1.2083)	17.4153	
	Aubrun et al., 2008	90	2.6299 (2.0657; 3.1942)	17.3974	
	Hadi et al., 2010	30	−7.3485 (−9.3408; 5.3561)	16.2113	
	Guignard et al., 2002	50	−12.5382 (−15.0574; −10.0190)	15.5224	
	Yalcin et al., 2012	53	−10.4428 (−12.5024; −8.3831)	16.1292	
	Random effects	317	−4.0644 (−7.0110; −1.1178)		
Time to first rescue analgesia minor surgery	Van Elstraete, 2004	40	0.3305 (−0.2935; 0.9545)	34.1311	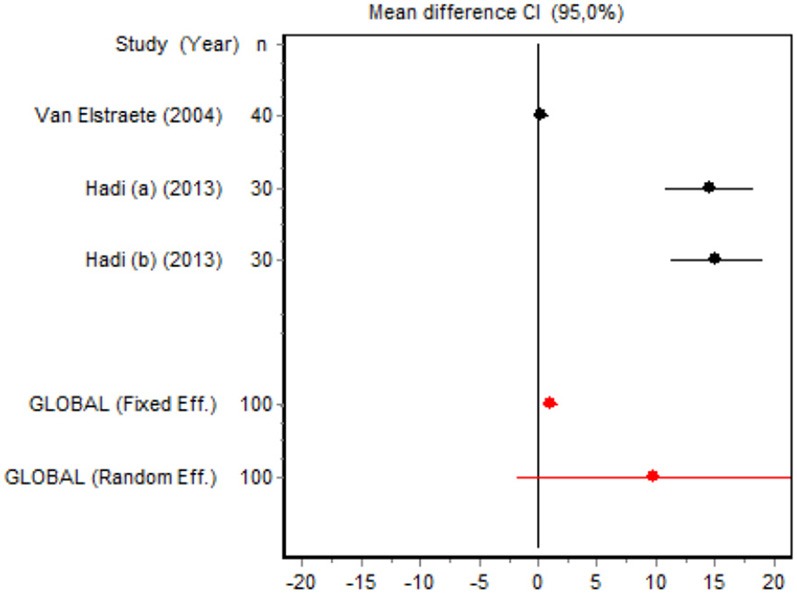
	Hadi et al., 2013	30	14.6007 (10.8376; 18.3638)	32.9752	
	Hadi et al., 2013	30	15.1306 (11.2358; 19.0254)	32.8937	
	Random effects	100	9.9044 (−1.6756; 214845)		
Time to first rescue analgesia mayor surgery	Jaksch et al., 2002	30	−1.2545 (−2.0374; −0.4716)	25.8794	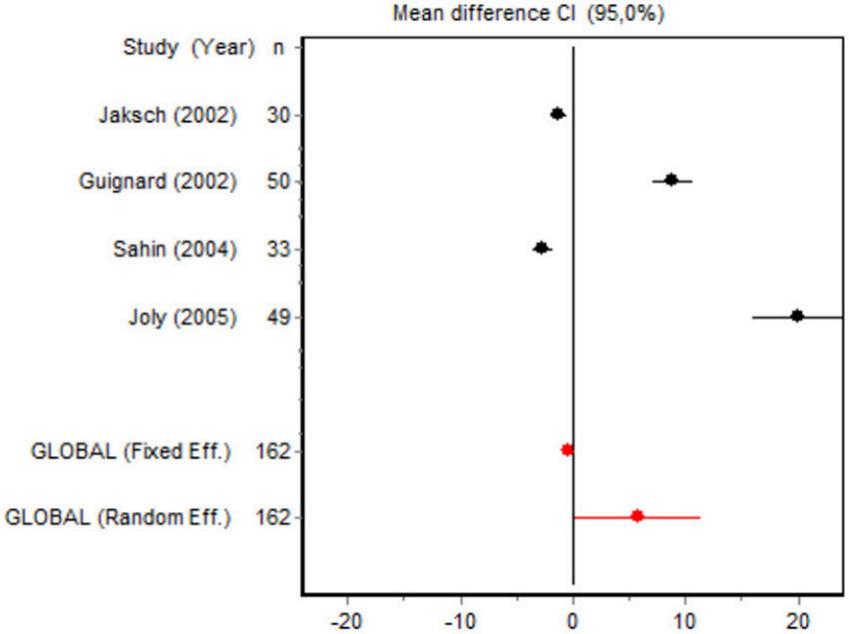
	Guignard et al., 2002	50	8.85487.0329; 10.6767	25.3153	
	Sahin et al., 2004	33	−2.6495 (−3.5847; −1.7143)	25.823	
	Joly et al., 2005	49	19.9587 (15.9676; 23.9497)	22.9816	
	Random effects	162	5.8196 (0.2130; 11.4261)		
Incidence of ponv minor surgery	Van Elstraete, 2004	40	1.3333 (0.3413; 5.2085)	15.8739	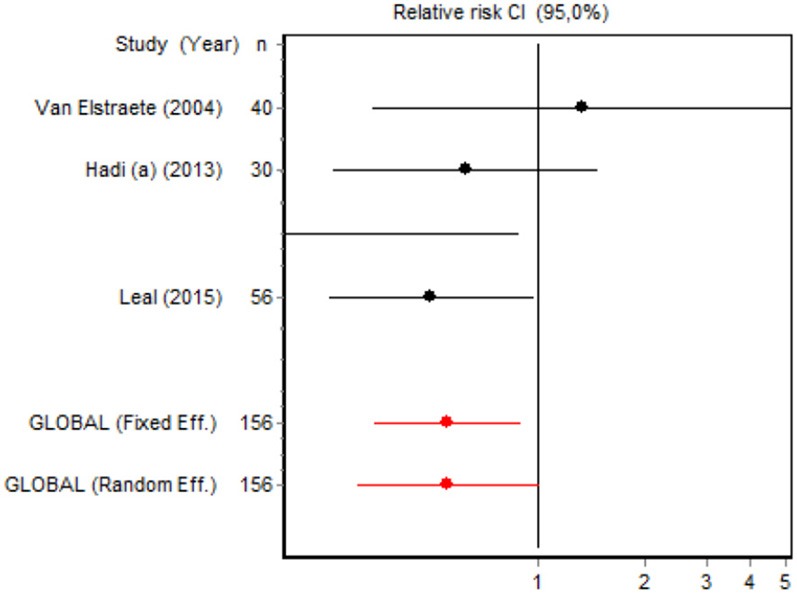
	Hadi et al., 2013	30	0.1250 (0.0178; 0.8802)	8.4422	
	Hadi et al., 2013	30	0.6250 (0.2650; 1.4741)	32.0743	
	Leal et al., 2015	56	0.5000 (0.2564; 0.9749)	43.6096	
	Random effects	156	0.5583 (0.3084; 1.0105)		
Incidence of ponv major surgery	Guignard et al., 2002	50	0.80008 (0.2428; 2.6355)	7.0831	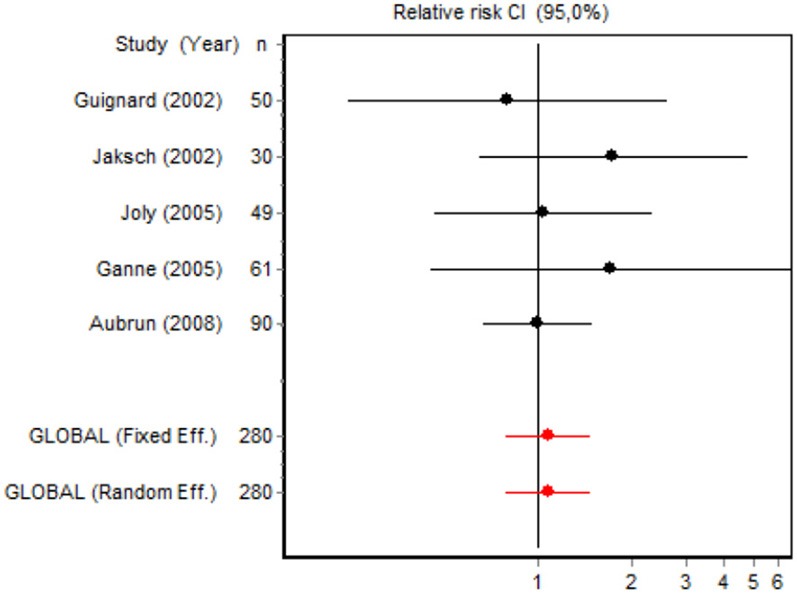
	Jaksch et al., 2002	30	1.7500 (0.6448; 4.7497)	10.0983	
	Joly et al., 2005	49	1.0417 (0.4661; 2.3279)	15.5688	
	Ganne et al., 2005	61	1.7222 (0.4508; 6.5802)	5.6030	
	Aubrun et al., 2008	90	1.0000 (0.6676; 1.4980)	61.6469	
	Fixed effects	280	1.0806 (0.7868; 1.4841)		
Incidence of psycotic events major surgery	Yalcin et al., 2012	30	1.0000 (0.0687; 14.553)	20.7547	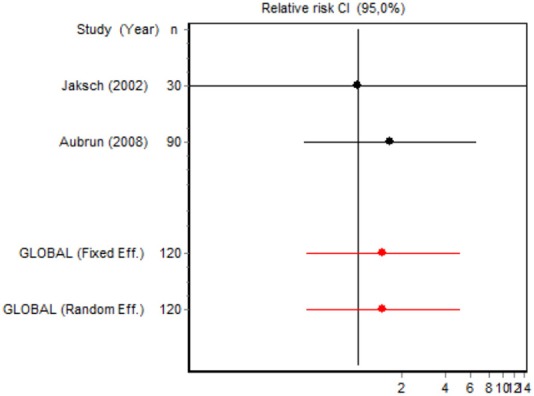
	Aubrun et al., 2008	90	1.6667 (0.4233; 6.5617)	79.2453	
	Fixed effects	120	1.4990 (0.4426; 5.0771)		

*PONV, Postoperative nausea and vomiting; RR, Risk ratio. Subgroup 15 was excluded from the meta-analysis*.

#### Cumulative morphine consumption

Nine of the 12 RCTs reported cumulative morphine consumption as an outcome measure for assessing ketamine efficacy at 24 h. The data were heterogeneous in the minor and major surgery groups (*p* < 0.001). As indicated by the forest plot (Figure 1), morphine consumption in the first 24 h was significantly lower in the intervention group than in the control group in patients who underwent both minor surgery (SMD = −8.3099, 95% CI: −12.0904 to −4.5295) and major surgery (SMD = −4.0644, 95% CI: −7.0110 to −1,1178). In the case of minor surgery, the sensitivity analysis showed that the elimination of the studies by Van Elstraete et al. and Leal et al. would moderately change the global effect and lead to larger CI, but it would not change the statistical significance and the conclusions. In major surgery, only the elimination of the study by Guignard et al. (2002) would lead to a loss of statistical significance (Annex 2 in Supplementary Material).

#### Postoperative pain intensity

Eight RCTs involving 475 patients reported data on pain intensity at rest in the first 24 postoperative hours. The data were heterogeneous at all points of follow-up except for the first 2 h in the minor surgery group. The forest plots showed a significant decrease in pain intensity with ketamine and morphine compared with morphine only in the minor surgery group at 2, 12, and 24 h and in the major surgery group at 2 h (Table 4). Pain intensity in the first 24 h was significantly lower with ketamine in the minor surgery group (SMD = −5.7507, 95% CI: −10.8028 to −0.6986) but not in the major surgery group (Table 4). According to the sensitivity analysis, removing the studies by Van Elstraete et al. and Hadi et al. would lead to a loss of statistical significance in some cases. On the other hand, the elimination of the study by Ganne et al. in the case of pain intensity after 4 h in major surgery would make the result statistically significant, showing favorable results for the ketamine group (Annex 2 in Supplementary Material).

#### Time to first rescue analgesia

Six RCTs involving 262 patients reported data on time of the first request for analgesia in the postoperative period. There were three RCTs (100 patients) in the minor surgery group and four (162 patients) in the major surgery group, and both groups were affected by significant heterogeneity (*p* < 0.001). The forest plot shows a significantly longer time to the first rescue analgesia for ketamine in the major surgery group (SMD = 5.8196, 95% CI: 0.2130–11.4261) but not in the minor surgery group (Table 4). However, if the study by Van Elstraete et al. would be removed, the results of minor surgery would be statistically significant (Annex 2 in Supplementary Material).

#### Adverse effects

Eight RCTs involving 326 patients reported on PONV, while two involving 120 patients reported on psychotic events (Table 4). According to the pooled analysis, ketamine administration was not a protective or risk factor for the occurrence of adverse effects, since all the CIs for the overall measure contained the value RR = 1, indicating no differences between the intervention and the control group.

## Discussion

Postoperative opioid-induced hyperalgesia can have significant clinical consequences, including inadequate pain control, increased opioid consumption, and a greater risk of adverse effects, ultimately leading to higher morbidity, longer hospital stays, and a greater likelihood of chronic postsurgical pain (Fletcher and Martinez, 2014). Opioid-induced hyperalgesia is believed to be due to changes in the central and peripheral nervous systems that result in sensitization of the pronociceptive pathways. Numerous mechanisms have been implicated in the pathophysiology of this condition, notably those involving the central glutamatergic system, since the NMDA receptor antagonism prevents the opioid-induced pain sensitivity and the perturbation of spinal glutamate transporter activity modulates the development of opioid-induced hyperalgesia. The activation of spinal dynorphin content, and the increase in the evoked release of spinal excitatory neuropeptides such as calcitonin gene-related peptide from primary afferents is also evoked. Other phenomena involved are related to neuroplastic changes in the rostral ventromedial medulla that would increase the activity of the facilitating descending nociceptive pathways. In addition, peripheral sensitization involving the activity of protein kinase C is also involved (Chu et al., 2008). The existence of multifactorial pathogenic features could explain the conflicting results reported for the efficacy of ketamine to date, as this anesthetic alone may not be able to block central sensitization and prevent opioid-induced hyperalgesia (Roeckel et al., 2016).

Low remifentanil doses (≥0.1 μg/kg/min or ≥2.7 ng/ml) appear to be sufficient for inducing hyperalgesia (Kim et al., 2014). We analyzed 12 RCTs comparing remifentanil-based general anesthesia (with doses ranging from 0.01 to 0.5 μg/kg/min or 2–4 ng/ml) with and without low-dose ketamine (infusion <1.2 mg/kg/h or bolus injection <1 mg/kg) (Kim et al., 2014).

Our systematic review is the first to analyze the effects of ketamine sedation according to the type of surgery (major vs. minor). Minor surgical procedures were defined as procedures that required a minimum hospital stay, such as arthroscopy, laparoscopy, and microsurgery. Although greater pain intensity might be expected after major surgery due to the size of the surgical field, the intensity of the nociceptive stimuli, and the longer operative times, a high incidence of postoperative pain has also been reported for laparoscopic and other minor procedures (Gerbershagen et al., 2013). In our study, the favorable results observed for ketamine vs. no ketamine in the first 2, 12, and 24 h in the minor surgery group (Table 4) may be related to the fact that lower doses of analgesia are used for minor procedures and they may have been insufficient to relieve postoperative pain in the control group.

Remifentanil-based general anesthesia does not offer sufficient guarantees for adequate postoperative pain management in major or minor surgery without the application of multimodal analgesic **regimens** adapted to each procedure. If the necessary analgesic effect is not achieved, activation of NMDA receptors during surgery could give rise to an erroneous interpretation of results (Van Elstraete, 2004).

Our findings show that perioperative ketamine was associated with a significant reduction in the consumption of morphine 24 h after minor and major surgery. Conflicting results have been reported in the literature. In a meta-analysis of RCTs involving 649 adults and children and adults who underwent spine surgery, Pendi et al. (2018) reported that perioperative ketamine significantly reduced morphine consumption and pain intensity. However, another meta-analysis of 11 studies examining the use of ketamine in children did not find any significant reduction in morphine consumption. The differences could be due to different pharmacokinetic profiles in children and adults or to variations in anesthetic regimens and pain scales (Michelet et al., 2016).

Our findings on morphine consumption should be interpreted with caution, as the publication bias analysis showed a risk of bias in both the minor and major surgery subgroups. Although the results for some of the subgroups could have been improved by removing certain studies, we did not do this because this would have introduced an additional source of bias and because the sensitivity analysis supported the robustness of the meta-analysis results (data not shown).

Apart from exerting a morphine-sparing effect, ketamine also significantly reduced pain intensity in the early postoperative period after major and minor surgery, supporting results from previous meta-analyses of NMDA receptor antagonists, including magnesium sulfate (Liu et al., 2012; Wu et al., 2015). The effects of remifentanil-induced hyperalgesia appear to be greatest during this early postoperative period (Fletcher and Martinez, 2014) and ketamine may help to reduce pain at this time because it provides residual analgesia in relation to its relatively rapid clearance (890–1,227 mL/min) and short elimination half-life (2–3 h) (Mion and Villevieille, 2013).

In their meta-analysis of 14 RCTs involving 623 patients, Wu et al. (2015) observed no differences in morphine consumption or time to first rescue analgesia between patients who received an NMDA receptor (ketamine or magnesium sulfate) and controls. Conflicting findings from meta-analyses on the efficacy of ketamine in preventing remifentanil-induced hyperalgesia have been attributed to the inclusion of RCTs examining several NMDA antagonists (Liu et al., 2012). In order to avoid that, we decided to minimize sources of variability by excluding studies of all NMDA receptor antagonists other than ketamine. The variations observed in the anesthesia and analgesia protocols in the RCTs included in our systematic review can be explained by the fact that the studies were from six countries (Table 2).

Conceptually, general anesthesia can be maintained during surgery by total intravenous anesthesia (TIVA) or balanced anesthesia (inhalation of volatile agents). Four of the RCTs in our study used TIVA (with propofol) (Jaksch et al., 2002; Van Elstraete et al., 2004; Ganne et al., 2005) while eight used volatile agents (Schmid et al., 1999; Sahin et al., 2004; Joly et al., 2005; Hadi et al., 2010). Propofol has traditionally been considered to be a hypnotic sedative without analgesic properties, although there is evidence that it might have a modulatory effect on nociceptive processing and perception (Bandschapp et al., 2010), reflected in the observation of less intense postoperative pain compared with general balanced anesthesia (Cheng et al., 2008). The potential modulatory role of propofol in opioid-induced hyperalgesia may be due to its interaction with GABA-A receptors at the supraspinal level (Wang et al., 2004; Singler et al., 2007), to its non-competitive inhibition of NMDA (in particular the NR1 subunit) (Orser et al., 1995; Kingston et al., 2006), or to its neuroprotective effects (demonstrated in animal models) (Grasshoff and Gillessen, 2005). Propofol could reduce hyperalgesia induced by high doses of remifentanil during maintenance of intravenous anesthesia (Shin et al., 2010) and consequently improve postoperative outcomes and consumption of morphine. As shown by the influence graph for pain intensity in the minor surgery group, only one of the studies would have substantially modified the overall result had it been eliminated from the meta-analysis and this was the propofol-based anesthesia article by Van Elstraete et al. (2004). Its removal would have eliminated the statistical difference between the ketamine and control groups at 30 min, 1, 12, and 24 h, generating inconclusive results.

We found no significant differences between patients who received preemptive ketamine and those who did not for opioid-related adverse effects or ketamine-related psychotomimetic effects. Our findings thus support previous findings (Assouline et al., 2016) that subanesthetic doses of perioperative ketamine are safe.

The findings of this systematic review and meta-analysis show that, when used as an adjuvant to morphine, ketamine reduces postoperative morphine consumption and pain intensity in the early postoperative period in adults undergoing major and minor surgery. Our study has some limitations, including (1) possible confounding by the high prevalence of postoperative pain; (2) the variability of anesthetic regimens and study populations together with the individual variability reflected in the different subgroups; (3) the multifactorial pathogenesis of opioid-induced hyperalgesia together with the lack of a protocol for the objective measurement of different types of hyperalgesia; and (4) the lack of standardized, clearly defined scales to measure postoperative pain, morphine consumption, and ketamine- and opioid-related adverse effects. These limitations should be taken into account when designing future RCTs.

## Conclusions

In summary, our systematic review and meta-analysis provide evidence that subanesthetic intraoperative doses of ketamine have a beneficial effect on pain control in the immediate postoperative period (24 h), as they reduce the consumption of postoperative morphine and the intensity of pain following minor and major surgery. The addition of low doses of ketamine to remifentanil-based general anesthesia regimens should be considered.

While our findings may help to explain some of the conflicting evidence on the use of preemptive ketamine, postoperative pain management remains a challenge and further research using standardized protocols and scales is needed.

## Author contributions

JG-H and JM-M conceived and designed the study. JG-H, JM-M, and AS participated in study selection and data extraction. AS performed statistical analysis. JG-H, JM-M, AS, and EP were involved in manuscript drafting and revision. All authors approved the final manuscript for submission and publication.

### Conflict of interest statement

The authors declare that the research was conducted in the absence of any commercial or financial relationships that could be construed as a potential conflict of interest. The reviewer DS and handling Editor declared their shared affiliation.

## References

[B1] Angst M. S. Clark J. D. (2006). Opioid-induced hyperalgesia: a qualitative systematic review. Anesthesiology 104, 570–587. 10.1097/00000542-200603000-00025 16508405

[B2] Assouline B. Tramèr M. R. Kreienbühl L. Elia N. (2016). Benefit and harm of adding ketamine to an opioid in a patient-controlled analgesia device for the control of postoperative pain: systematic review and meta-analyses of randomized controlled trials with trial sequential analyses. Pain 157, 2854–2864. 10.1097/j.pain.0000000000000705 27780181

[B3] Aubrun F. Gaillat C. Rosenthal D. Dupuis M. Mottet P. Marchetti F.. (2008). Effect of a low-dose ketamine regimen on pain, mood, cognitive function and memory after major gynaecological surgery. Eur. J. Anaesthesiol. 25, 97–105. 10.1017/S0265021507002566 17894912

[B4] Bandschapp O. Filitz J. Ihmsen H. Berset A. Urwyler A. Koppert W.. (2010). Analgesic and antihyperalgesic properties of propofol in a human pain model. Anesthesiology 113, 421–428. 10.1097/ALN.0b013e3181e33ac8 20613472

[B5] Bell R. F. Dahl J. B. Moore R. A. Kalso E. A. (2015). Perioperative ketamine for acute postoperative pain. Cochrane Database Syst. Rev. CD004603. 10.1002/14651858.CD004603.pub3 16437490

[B6] Carstensen M. Möller A. M. (2010). Adding ketamine to morphine for intravenous patient-controlled analgesia for acute postoperative pain: a qualitative review of randomized trials. Br. J. Anaesth. 104, 401–406. 10.1093/bja/aeq041 20207747

[B7] Cheng S. S. Yeh J. Flood P. (2008). Anesthesia matters: patients anesthetized with propofol have less postoperative pain than those anesthetized with isoflurane. Anesth. Analg. 106, 264–269. 10.1213/01.ane.0000287653.77372.d9 18165589

[B8] Choi E. Lee H. Park H. S. Lee G. Y. Kim Y. J. Baik H.-J. (2015). Effect of intraoperative infusion of ketamine on remifentanil-induced hyperalgesia. Korean J. Anesthesiol. 68, 476–480. 10.4097/kjae.2015.68.5.476 26495058 PMC4610927

[B9] Chou R. Gordon D. B. de Leon-Casasola O. A. Rosenberg J. M. Bickler S. Brennan T.. (2016). Management of postoperative pain: a clinical practice guideline from the American pain society, the American society of regional anesthesia and pain medicine, and the American society of anesthesiologists' committee on regional anesthesia, executive committee, and administrative council. J. Pain 17, 131–157. 10.1016/j.jpain.2015.12.008 26827847

[B10] Chu L. F. Angst M. S. Clark D. (2008). Opioid-induced hyperalgesia in humans: molecular mechanisms and clinical considerations. Clin. J. Pain 24, 479–496. 10.1097/AJP.0b013e31816b2f43 18574358

[B11] Clark H. D. Wells G. A. Huët C. McAlister F. A. Salmi L. R. Fergusson D.. (1999). Assessing the quality of randomized trials: reliability of the Jadad scale. Control. Clin. Trials 20, 448–452. 10.1016/S0197-2456(99)00026-4 10503804

[B12] Fletcher D. Martinez V. (2014). Opioid-induced hyperalgesia in patients after surgery: a systematic review and a meta-analysis. Br. J. Anaesth. 112, 991–1004. 10.1093/bja/aeu137 24829420

[B13] Fletcher D. Stamer U. M Pogatzki-Zahn E. Zaslansky R. Tanase N. V. Perruchoud C. (2015). Chronic postsurgical pain in Europe: an observational study. Eur. J. Anaesthesiol. 32, 725–734. 10.1097/EJA.0000000000000319 26241763

[B14] Ganne O. Abisseror M. Menault P. Malhière S. Chambost V. Charpiat B.. (2005). Low-dose ketamine failed to spare morphine after a remifentanil-based anaesthesia for ear, nose and throat surgery. Eur. J. Anaesthesiol. 22, 426–430. 10.1017/S0265021505000724 15991504

[B15] Gerbershagen H. J. Aduckathil S. van Wijck A. J. M. Peelen L. M. Kalkman C. J. Meissner W. (2013). Pain intensity on the first day after surgery. anesthesiology. Am. Soc. Anesthesiol. 118, 934–44. 10.1097/ALN.0b013e31828866b3 23392233

[B16] Gordt K. Gerhardy T. Najafi B. Schwenk M. (2018). Effects of wearable sensor-based balance and gait training on balance, gait, and functional performance in healthy and patient populations: a systematic review and meta-analysis of randomized controlled trials. Gerontology 64, 74–89. 10.1159/000481454 29130977

[B17] Grasshoff C. Gillessen T. (2005). Effects of propofol on N-methyl-D-aspartate receptor-mediated calcium increase in cultured rat cerebrocortical neurons. Eur. J. Anaesthesiol. 22, 467–470. 10.1017/S0265021505000803 15991512

[B18] Guignard B. Coste C. Costes H. Sessler D. I. Lebrault C. Morris W.. (2002). Supplementing desflurane-remifentanil anesthesia with small-dose ketamine reduces perioperative opioid analgesic requirements. Anesth. Analg. 95, 103–108. 10.1097/00000539-200207000-00018 12088951

[B19] Hadi B. A. Daas R. Zelkó R. (2013). A randomized, controlled trial of a clinical pharmacist intervention in microdiscectomy surgery - low dose intravenous ketamine as an adjunct to standard therapy. Saudi Pharm. J. 21, 169–175. 10.1016/j.jsps.2012.08.002 23960832 PMC3744926

[B20] Hadi B. A. Ramadani R. Daas R. Naylor I. Zelkó R. (2010). Remifentanil in combination with ketamine versus remifentanil in spinal fusion surgery – a double blind study. Int. J. Clin. Pharmacol. Ther. 48, 542–548. 10.5414/CPP48542 20650046

[B21] Hong B. H. Lee W. Y. Kim Y. H. Yoon S. H. Lee W. H. (2011). Effects of intraoperative low dose ketamine on remifentanil-induced hyperalgesia in gynecologic surgery with sevoflurane anesthesia. Korean J. Anesthesiol. 61, 238–243. 10.4097/kjae.2011.61.3.238 22025947 PMC3198186

[B22] Jaksch W. Lang S. Reichhalter R. Raab G. Dann K. Fitzal S. (2002). Perioperative small-dose S(+)-ketamine has no incremental beneficial effects on postoperative pain when standard-practice opioid infusions are used. Anesth. Analg. 94, 981–986. 10.1097/00000539-200204000-00038 11916808

[B23] Joly V. Richebe P. Guignard B. Fletcher D. Maurette P. Sessler D. I.. (2005). Remifentanil-induced postoperative hyperalgesia and its prevention with small-dose ketamine. Anesthesiology 103, 147–155. 10.1097/00000542-200507000-00022 15983467

[B24] Kang J. Chang J. Y. Sun X. Men Y. Zeng H. Hui Z. (2018). Role of postoperative concurrent chemoradiotherapy for esophageal carcinoma: a meta-analysis of patients. J. Cancer 9, 584–593. 10.7150/jca.20940 29483964 PMC5820926

[B25] Kim S. H. Stoicea N. Soghomonyan S. Bergese S. D. (2014). Intraoperative use of remifentanil and opioid induced hyperalgesia/acute opioid tolerance: systematic review. Front. Pharmacol. 5:108. 10.3389/fphar.2014.00108 24847273 PMC4021143

[B26] Kim S. H. Stoicea N. Soghomonyan S. Bergese S. D. (2015). Remifentanil-acute opioid tolerance and opioid-induced hyperalgesia: a systematic review. Am. J. Ther. 22, 62–74. 10.1097/MJT.0000000000000019 25830866

[B27] Kingston S. Mao L. Yang L. Arora A. Fibuch E. E. Wang J. Q. (2006). Propofol inhibits phosphorylation of N-methyl-D-aspartate receptor NR1 subunits in neurons. Anesthesiology 104, 763–769. 10.1097/00000542-200604000-00021 16571972

[B28] Kohrs R. Durieux M. E. (1998). Ketamine: teaching an old drug new tricks. Anesth. Analg. 87, 1186–1193. 9806706 10.1097/00000539-199811000-00039

[B29] Launo C. Bassi C. Spagnolo L. Badano S. Ricci C. Lizzi A.. (2004). Preemptive ketamine during general anesthesia for postoperative analgesia in patients undergoing laparoscopic cholecystectomy. Minerva Anestesiol. 70, 727–738. 15516884

[B30] Leal P. C. Salomão R. Brunialti M. K. Sakata R. K. (2015). Evaluation of the effect of ketamine on remifentanil-induced hyperalgesia: a double-blind, randomized study. J. Clin. Anesth. 27, 331–337. 10.1016/j.jclinane.2015.02.002 25910532

[B31] Lee M. H. Chung M. H. Han C. S. Lee J. H. Choi Y. R. Choi E. M.. (2014). Comparison of effects of intraoperative esmolol and ketamine infusion on acute postoperative pain after remifentanil-based anesthesia in patients undergoing laparoscopic cholecystectomy. Korean J. Anesthesiol. 66, 222–228. 10.4097/kjae.2014.66.3.222 24729845 PMC3983419

[B32] Liu Y. Zheng Y. Gu X. Ma Z. (2012). The efficacy of NMDA receptor antagonists for preventing remifentanil-induced increase in postoperative pain and analgesic requirement: a meta-analysis. Minerva Anestesiol. 78, 653–667. 22301767

[B33] Maher C. G. Sherrington C. Herbert R. D. Moseley A. M. Elkins M. (2003). Reliability of the PEDro scale for rating quality of randomized controlled trials. Phys. Ther. 83, 713–721. 10.1093/ptj/83.8.713 12882612

[B34] Mao J. (2006). Opioid-induced abnormal pain sensitivity. Curr. Pain Headache Rep. 10, 67–70. 10.1007/s11916-006-0011-5 16499832

[B35] Mao J. Price D. D. Mayer D. J. (1995). Mechanisms of hyperalgesia and morphine tolerance: a current view of their possible interactions. Pain 62, 259–274. 10.1016/0304-3959(95)00073-2 8657426

[B36] Mayer D. J. Mao J. Holt J. Price D. D. (1999). Cellular mechanisms of neuropathic pain, morphine tolerance, and their interactions. Proc. Natl Acad. Sci. U.S.A. 96, 7731–7736. 10.1073/pnas.96.14.7731 10393889 PMC33610

[B37] Michelet D. Hilly J. Skhiri A. Abdat R. Diallo T. Brasher C.. (2016). Opioid-sparing effect of ketamine in children: a meta-analysis and trial sequential analysis of published studies. Paediatr. Drugs 18, 421–433. 10.1007/s40272-016-0196-y 27688125

[B38] Michelet P. Guervilly C. Helaine A. Avaro J. P. Blayac D. Gaillat F.. (2007). Adding ketamine to morphine for patient-controlled analgesia after thoracic surgery: influence on morphine consumption, respiratory function, and nocturnal desaturation. Br. J. Anaesth. 99, 396–403. 10.1093/bja/aem168 17576969

[B39] Mion G. Villevieille T. (2013). Ketamine pharmacology: an update (pharmacodynamics and molecular aspects, recent findings). CNS Neurosci. Ther. 19, 370–380. 10.1111/cns.12099 23575437 PMC6493357

[B40] Moher D. Pham B. Jones A. Cook D. J. Jadad A. R. Moher M.. (1998). Does quality of reports of randomised trials affect estimates of intervention efficacy reported in meta-analyses? Lancet 352, 609–613. 10.1016/S0140-6736(98)01085-X 9746022

[B41] Orser B. A. Bertlik M. Wang L. Y. MacDonald J. F. (1995). Inhibition by propofol (2,6 di-isopropylphenol) of the N-methyl-D-aspartate subtype of glutamate receptor in cultured hippocampal neurones. Br. J. Pharmacol. 116, 1761–1768. 10.1111/j.1476-5381.1995.tb16660.x 8528557 PMC1909100

[B42] Ossipov M. H. Lai J. King T. Vanderah T. W. Porreca F. (2005). Underlying mechanisms of pronociceptive consequences of prolonged morphine exposure. Biopolymers 80, 319–324. 10.1002/bip.20254 15795927

[B43] Pendi A. Field R. Farhan S. D. Eichler M. Bederman S. S. (2018). Perioperative ketamine for analgesia in spine surgery: a meta-analysis of randomized controlled trials. Spine 43, E299–E307. 10.1097/BRS.0000000000002318 28700455 PMC5846492

[B44] Perkins F. M. Kehlet H. (2000). Chronic pain as an outcome of surgery. A review of predictive factors. Anesthesiology 93, 1123–1133. 10.1097/00000542-200010000-00038 11020770

[B45] Pozek J. J. Beausang D. Baratta J. L. Viscusi E. R. (2016). The acute to chronic pain transition: can chronic pain be prevented? Med. Clin. North Am. 100, 17–30. 10.1016/j.mcna.2015.08.005 26614716

[B46] Reddi D. (2016). Preventing chronic postoperative pain. Anaesthesia 71(Suppl. 1), 64–71. 10.1111/anae.13306 26620149

[B47] Roeckel L. A. Le Coz G. M. Gavériaux-Ruff C. Simonin F. (2016). Opioid-induced hyperalgesia: cellular and molecular mechanisms. Neuroscience 338, 160–182. 10.1016/j.neuroscience.2016.06.029 27346146

[B48] Sahin A. Canbay O. Cuhadar A. Celebi N. Aypar U. (2004). Bolus ketamine does not decrease hyperalgesia after remifentanil infusion. Pain Clin. 16, 407–411. 10.1163/1568569042664413

[B49] Schmid R. L. Sandler A. N. Katz J. (1999). Use and efficacy of low-dose ketamine in the management of acute postoperative pain: a review of current techniques and outcomes. Pain 82, 111–125. 10.1016/S0304-3959(99)00044-5 10467917

[B50] Shin S. W. Cho A. R. Lee H. J. Kim H. J. Byeon G. J. Yoon J. W.. (2010). Maintenance anaesthetics during remifentanil-based anaesthesia might affect postoperative pain control after breast cancer surgery. Br. J. Anaesth. 105, 661–667. 10.1093/bja/aeq257 20876698

[B51] Shipton E. A. (2014). The transition of acute postoperative pain to chronic pain: Part 1 - Risk factors for the development of postoperative acute persistent pain. Trends Anaesth. Crit. Care 4, 67–70. 10.1016/j.tacc.2014.04.001

[B52] Singler B. Troster A. Manering N. Schüttler J. Koppert W. (2007). Modulation of Remifentanil-Induced Postinfusion Hyperalgesia by Propofol. Anesthesia & Analgesia 104, 1397–1403. 10.1213/01.ane.0000261305.22324.f3 17513631

[B53] Song J. W. Shim J. K. Song Y. Yang S. Y. Park S. J. Kwak Y. L. (2013). Effect of ketamine as an adjunct to intravenous patient-controlled analgesia, in patients at high risk of postoperative nausea and vomiting undergoing lumbar spinal surgery. Br. J. Anaesth. 111, 630–635. 10.1093/bja/aet192 23744819

[B54] Van Elstraete A. C. (2004). Are preemptive analgesic effects of ketamine linked to inadequate perioperative analgesia. Anesth. Analg. 99:1576 10.1213/01.ANE.0000137441.79168.C5 15502071

[B55] Van Elstraete A. C. Lebrun T. Sandefo I. Polin B. (2004). Ketamine does not decrease postoperative pain after remifentanil-based anaesthesia for tonsillectomy in adults. Acta Anaesthesiol. Scand. 48, 756–760. 10.1111/j.1399-6576.2004.00399.x 15196109

[B56] Wang Q. Y. Cao J. L. Zeng Y. M. Dai T. J. (2004). GABAA receptor partially mediated propofol-induced hyperalgesia at superspinal level and analgesia at spinal cord level in rats. Acta Pharmacol. Sin. 25, 1619–1625. 15569406

[B57] Williams J. T. Christie M. J. Manzoni O. (2001). Cellular and synaptic adaptations mediating opioid dependence. Physiol. Rev. 81, 299–343. 10.1152/physrev.2001.81.1.299 11152760

[B58] Wu L. Huang X. Sun L. (2015). The efficacy of N-methyl-D-aspartate receptor antagonists on improving the postoperative pain intensity and satisfaction after remifentanil-based anesthesia in adults: a meta-analysis. J. Clin. Anesth. 27, 311–324. 10.1016/j.jclinane.2015.03.020 25824051

[B59] Yalcin N. Uzun S. T. Reisli R. Borazan H. Otelcioglu S. (2012). A comparison of ketamine and paracetamol for preventing remifentanil induced hyperalgesia in patients undergoing total abdominal hysterectomy. Int. J. Med. Sci. 9, 327–333. 10.7150/ijms.4222 22745573 PMC3384914

[B60] Yu C. Luo Y. L. Xiao S. S. Li Y. Zhang Q. (2005). Comparison of the suppressive effects of tramadol and low-dose ketamine on the patients with postoperative hyperalgesia after remifentanil-based anaesthesia. West China J. Stomatol. 23, 404–406. 16285546

[B61] Zanos P. Moaddel R. Morris P. J. Riggs L. M. Highland J. N. Georgiou P.. (2018). Ketamine and ketamine metabolite pharmacology: insights into therapeutic mechanisms. Pharmacol. Rev. 70, 621–660. 10.1124/pr.117.015198 29945898 PMC6020109

