# Supplementary material for: Effects of Ketamine on Postoperative Pain After Remifentanil-Based Anesthesia for Major and Minor Surgery in Adults: A Systematic Review and Meta-Analysis
Source: Front Pharmacol. 2018 Aug 17;9:921. doi: 10.3389/fphar.2018.00921 (PMC6107835; doi:10.3389/fphar.2018.00921)
Supplement: Supplementary file 1 [file Table_1.DOCX]

Annex 1. Details of the risk of bias assessment.

| **Study** | **Year** | **Jadad score** | **PEDro score** | **Randomization** | **Blinding** | **Description of withdrawals** | **Allocation concealment** |
| --- | --- | --- | --- | --- | --- | --- | --- |
| Aubrun | 2008 | 5(5) | 11(11) | Random number table | Double blind | Yes | Sealed envelopes |
| Ganne | 2005 | 5(5) | 10(11) | Random number table | Double blind | Yes | Sealed envelopes |
| Guignard | 2012 | 5(5) | 10(11) | Computer-generated | Double blind | Yes | Unclear |
| Haidi | 2013 | 4(5) | 9(11) | Just mentioned random | Double blind | Yes | Unclear |
| Hadi | 2010 | 4(5) | 8(11) | Just mentioned random | Double blind | Yes | Unclear |
| Jaksch | 2002 | 4(5) | 9(11) | Just mentioned random | Double blind | Yes | Sealed envelopes |
| Joly | 2005 | 5(5) | 10(11) | Random number table | Double blind | Yes | Sealed envelopes |
| Leal, 2015 | 2015 | 5(5) | 10(11) | Computer-generated | Double blind | Yes | Sealed envelopes |
| Lee | 2014 | 3(5) | 8(11) | Computer-generated | Observer-random | Yes | Unclear |
| Sahin | 2004 | 4(5) | 9(11) | Just mentioned random | Double blind | Yes | Unclear |
| Van Elstraete, | 2004 | 4(5) | 9(11) | Random number table | Double blind | Yes | Unclear |
| Yalcin | 2012 | 3(5) | 9(11) | Computer-generated | Just mentioned blinded | Yes | Unclear |

Annex 2. Influence graphics.

| **Subgroup** | **Influence graphics** |
| --- | --- |
| **1: VAS 0-2H MINOR SURGERY** | 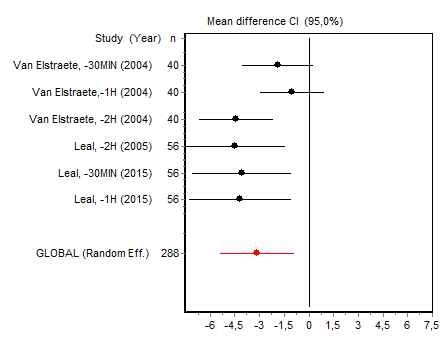 |
| **2:**  **VAS 0-2H MAJOR SURGERY** | 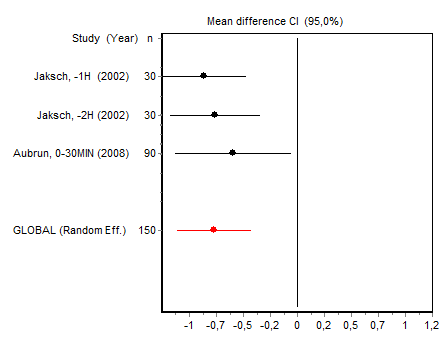 |
| **3: VAS 4H MINOR SURGERY** | * |
| **4: VAS 4H MAYOR SURGERY** | 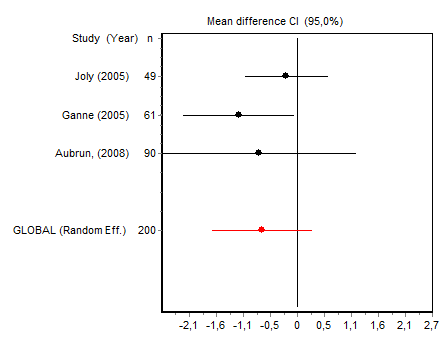 |
| **5: VAS 12H MINOR SURGERY** | 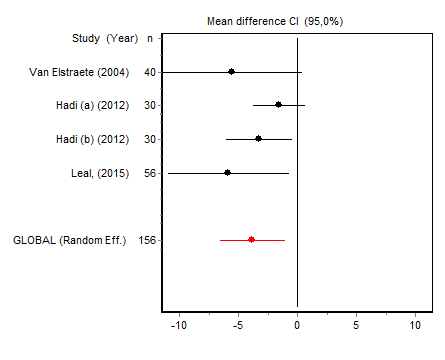 |
| **6: VAS 12H MAJOR SURGERY** | 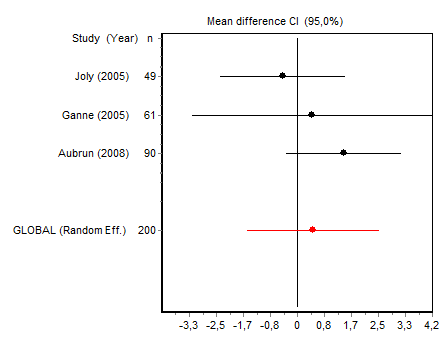 |
| **7: VAS 24H MINOR SURGERY** | 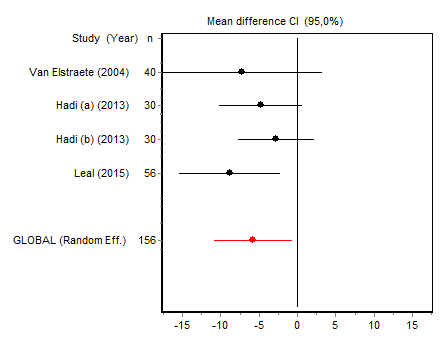 |
| **8: VAS 24H MAJOR SURGERY** | 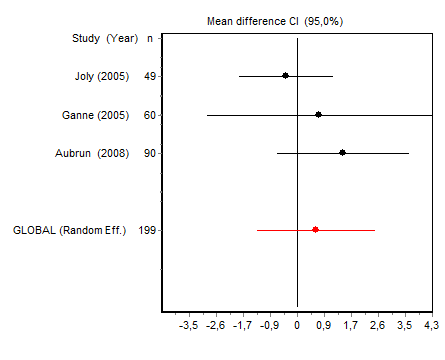 |
| **9: MORPHINE CONSUMPTION MAJOR SURGERY** | 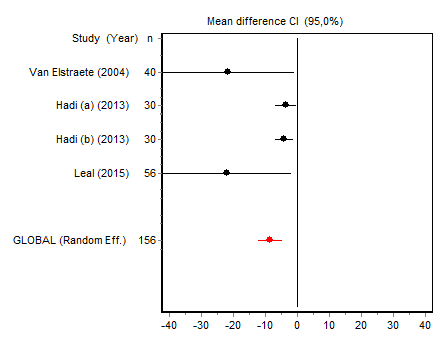 |
| **10: MORPHINE CONSUMPTION MAJOR SURGERY** | 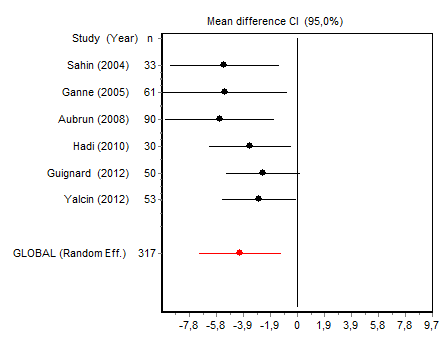 |
| **11: TIME TO FIRST RESCUE ANALGESIA**  **MINOR SURGERY** | 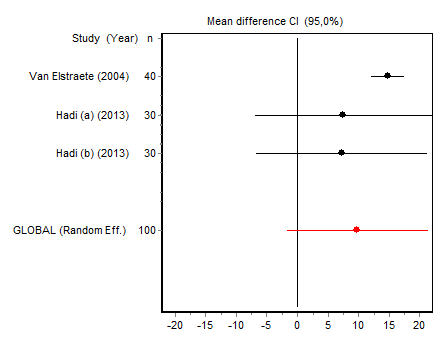 |
| **12: TIME TO FIRST RESCUE ANALGESIA MAYOR SURGERY** | 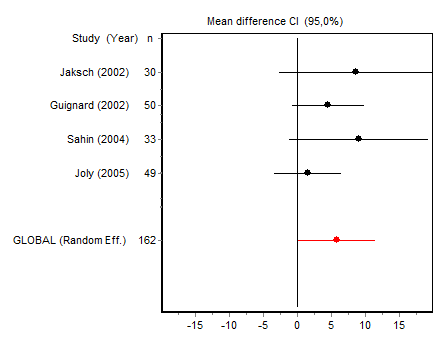 |
| **13: INCIDENCE OF PONV**  **MINOR SURGERY** | 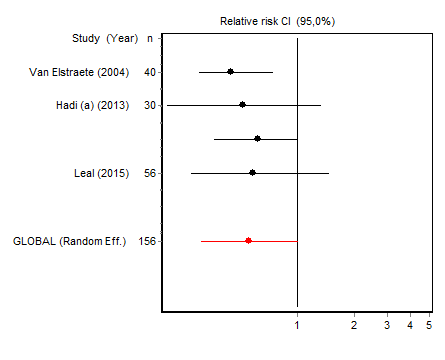 |
| **14: INCIDENCE OF PONV**  **MAJOR SURGERY** | 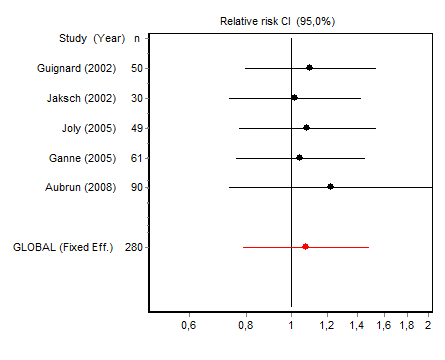 |
| **15: INCIDENCE OF PSYCOTIC EVENTS**  **MAJOR SURGERY** | * |
| *Results not shown for subgroups with only two studies. PONV, postoperative nausea and vomiting; VAS, visual analog scale.  ** Results not shown for subgroup 15, which was not included in the meta-analysis. | |
| **15** |  |
|  |  |
